# Estimators for Q_ST_ and coalescence times

**DOI:** 10.1002/ece3.2522

**Published:** 2016-10-05

**Authors:** Timothy D. Weaver

**Affiliations:** ^1^ Department of Anthropology University of California Davis CA USA

**Keywords:** $F_{\mathrm{ST}}$, genetic differentiation, neutral evolution, phenotype, quantitative genetics

## Abstract

Comparisons of $Q_{\mathrm{ST}}$ to $F_{\mathrm{ST}}$ can provide insights into the evolutionary processes that lead to differentiation, or lack thereof, among the phenotypes of different groups (e.g., populations, species), and these comparisons have been performed on a variety of taxa, including humans. Here, I show that for neutrally evolving (i.e., by genetic drift, mutation, and gene flow alone) quantitative characters, the two commonly used $Q_{\mathrm{ST}}$ estimators have somewhat different interpretations in terms of coalescence times, particularly when the number of groups that have been sampled is small. A similar situation occurs for $F_{\mathrm{ST}}$ estimators. Consequently, when observations come from only a small number of groups, which is not an unusual situation, it is important to match estimators appropriately when comparing $Q_{\mathrm{ST}}$ to $F_{\mathrm{ST}}$.

## 1 Introduction

An important goal of evolutionary studies is to understand the processes that lead to differentiation, or lack thereof, among the phenotypes of different groups (e.g., populations, species). We would like to know: did genetic drift or diversifying natural selection produce the between‐group differences? Or, in the case of limited differentiation, did stabilizing selection keep the phenotypes similar? One way to approach these questions is to compare the degree of genetic differentiation for phenotypes of interest, $Q_{\mathrm{ST}}$, with the degree of genetic differentiation for presumably neutral DNA markers, $F_{\mathrm{ST}}$ (Prout & Barker, 1993; Relethford, 1994; Rogers & Harpending, 1983; Spitze, 1993). These kinds of comparisons have been performed for numerous taxa (reviewed by Whitlock, 2008), including humans (reviewed by Roseman & Weaver, 2007).

## 2 Different $Q_{\mathrm{ST}}$ Estimators

Two $Q_{\mathrm{ST}}$ estimators for quantitative characters are commonly used. Prout and Barker (1993) and Spitze (1993) defined $Q_{\mathrm{ST}}$ as

$$Q_{\mathrm{ST}}=\frac{V_{A,B}}{V_{A,B}+2V_{A,W}}$$

where $V_{A,B}$ is the between‐group additive genetic variance and $V_{A,W}$ is the within‐group additive genetic variance of the character of interest (see also Lande, 1992). This definition implies the $Q_{\mathrm{ST}}$ estimator

(1) $${\hat{Q}}_{\mathrm{ST}}^{\mathrm{PBS}}=\frac{{\hat{V}}_{A,B}}{{\hat{V}}_{A,B}+2{\hat{V}}_{A,W}}$$

where the hats on the variances denote unbiased estimators (see Prout & Barker, 1993; Spitze, 1993; Whitlock, 2008).

Relethford and Blangero (1990) and Relethford (1994) introduced a different $Q_{\mathrm{ST}}$ estimator. For a single character with equal weighting of groups, this estimator reduces to

(2) $${\hat{Q}}_{\mathrm{ST}}^{\mathrm{RB}}=\frac{1}{d}\sum_{i=1}^{d}\frac{{\left({\overset{¯}{z}}_{i}-\overset{¯}{z}\right)}^{2}}{h^{2}V_{P,W}}/\left(2+\frac{1}{d}\sum_{i=1}^{d}\frac{{\left({\overset{¯}{z}}_{i}-\overset{¯}{z}\right)}^{2}}{h^{2}V_{P,W}}\right)$$

where *d* is the number of groups that have been sampled, ${\overset{¯}{z}}_{i}$ is the character mean for the *i*th group, $\overset{¯}{z}$ is the character grand mean (mean of the group means), $h^{2}$ is the narrow‐sense heritability of the character, and $V_{P,W}$ is the within‐group phenotypic variance of the character (see also Rogers & Harpending, 1983). By multiplying Equation (2) by $h^{2}V_{P,W}/h^{2}V_{P,W}$, it is straightforward to show that ${\hat{Q}}_{\mathrm{ST}}^{\mathrm{RB}}$ can be defined alternatively as

(3) $${\hat{Q}}_{\mathrm{ST}}^{\mathrm{RB}}=\frac{{\tilde{V}}_{A,B}}{{\tilde{V}}_{A,B}+2{\hat{V}}_{A,W}}$$

where ${\tilde{V}}_{A,B}$ is the biased estimator (i.e., division by *d* instead of *d*−1) for the between‐group additive genetic variance, $V_{A,B}$. The limit of ${\hat{Q}}_{\mathrm{ST}}^{\mathrm{RB}}$ as the number of sampled groups goes to infinity is ${\hat{Q}}_{\mathrm{ST}}^{\mathrm{PBS}}$, but with a small number of sampled groups, these quantities can have quite different values. For example, when only two groups are sampled,

$${\hat{Q}}_{\mathrm{ST}}^{\mathrm{RB}(d=2)}=\frac{{\hat{V}}_{A,B}}{{\hat{V}}_{A,B}+4{\hat{V}}_{A,W}}.$$

Prout and Barker (1993) and Spitze (1993) does not cite Relethford and Blangero (1990), and Relethford (1994) does not cite Prout and Barker (1993) or Spitze (1993), so these two estimators were developed independently, and apparently, not many researchers are aware of both, with biologists citing Prout and Barker (1993) and Spitze (1993) and anthropologists citing Relethford and Blangero (1990) and Relethford (1994). Here, I show that for neutrally evolving (i.e., by genetic drift, mutation, and gene flow alone) characters, ${\hat{Q}}_{\mathrm{ST}}^{\mathrm{PBS}}$ and ${\hat{Q}}_{\mathrm{ST}}^{\mathrm{RB}}$ have somewhat different interpretations in terms of coalescence times.

## 3 Expressing $Q_{\mathrm{ST}}$ Estimators in Terms of Coalescence Times

Imagine sampling *d* groups, each for *n* individuals, and measuring a particular character on each individual. To proceed, we will start by expressing the problem in terms of breeding values for haplotypes and then relate these quantities to additive genetic variances. Let $S_{H,W}$ be the mean sum of squares of the differences in breeding values among the haplotypes of individuals from the same group, and $S_{H,B}$ be the mean sum of squares of the differences in breeding values among haplotypes of individuals from different groups. Then,

(4) $$\begin{array}{cc} S_{H,W} & =\frac{1}{d}\sum_{j=1}^{d}\frac{2}{2n(2n-1)}\sum_{i=1}^{2n-1}\sum_{i^{\prime }=i+1}^{2n}{\left(x_{i,j}-x_{i^{\prime },j}\right)}^{2}\;\text{and}\\ S_{H,B} & =\frac{2}{{\left(2n\right)}^{2}d\left(d-1\right)}\sum_{j=1}^{d-1}\sum_{j^{\prime }=j+1}^{d}\sum_{i=1}^{2n}\sum_{i^{\prime }=1}^{2n}{\left(x_{i,j}-x_{i^{\prime },j^{\prime }}\right)}^{2} \end{array}$$

where each *x* is a breeding value, *i* and $i^{\prime }$ index haplotypes, and *j* and $j^{\prime }$ index groups. Note that $S_{H,W}$ and $S_{H,B}$ quantify squared pairwise differences rather than squared deviations from a mean. If the character is evolving neutrally and its genetic basis is a large number of loci that contribute equally and additively to the value of the measurement (i.e., no interactions among the contributions), $S_{H,W}$ and $S_{H,B}$ can be expressed in terms of coalescence times as

(5) $$E\left\{S_{H,W}\right\}=4\tau_{W}\sigma_{m}^{2}\;\text{and}$$

(6) $$E\left\{S_{H,B}\right\}=4\tau_{B}\sigma_{m}^{2}$$

where E is the mathematical expectation (mean of the distribution of possible evolutionary outcomes), $\tau_{W}$ is the mean coalescence time of pairs of alleles from the same group, $\tau_{B}$ is the mean coalescence time of pairs of alleles from different groups, and $\sigma_{m}^{2}$ is the additive genetic variance introduced by mutation per zygote per generation into all of the groups (Slatkin, 1995; Whitlock, 1999). Additionally,

$$S_{H,W}=2{\hat{V}}_{H,W}$$

where ${\hat{V}}_{H,W}$ is the unbiased estimator for the variance in breeding values among the haplotypes of individuals from the same group (Slatkin, 1995). Furthermore, at Hardy–Weinberg equilibrium,

(7) $$S_{H,W}=4{\hat{V}}_{A,W}$$

because the variance in breeding values for the haplotypes of an individual is equal the within‐group additive genetic variance (Kremer, Zanetto, & Ducousso, 1997).

Let $x_{i,j}={\overset{¯}{x}}_{j}+\Delta_{i,j}$ where ${\overset{¯}{x}}_{j}$ is the mean breeding value for group *j* and $\Delta_{i,j}$ is the deviation of the breeding value of haplotype *i* of group *j* from the group mean. Then, substituting into Equation (4) gives

$$\begin{array}{cc} S_{H,B} & =\frac{2}{{\left(2n\right)}^{2}d\left(d-1\right)}\sum_{j=1}^{d-1}\sum_{j^{\prime }=j+1}^{d}\sum_{i=1}^{2n}\sum_{i^{\prime }=1}^{2n}{\left({\overset{¯}{x}}_{j}+\Delta_{i,j}-{\overset{¯}{x}}_{j^{\prime }}-\Delta_{i^{\prime },j^{\prime }}\right)}^{2}\\ & =\frac{2}{{\left(2n\right)}^{2}d\left(d-1\right)}\sum_{j=1}^{d-1}\sum_{j^{\prime }=j+1}^{d}\sum_{i=1}^{2n}\sum_{i^{\prime }=1}^{2n}\left({\left({\overset{¯}{x}}_{j}-{\overset{¯}{x}}_{j^{\prime }}\right)}^{2}+{\left(\Delta_{i,j}-\Delta_{i^{\prime },j^{\prime }}\right)}^{2}+2\left({\overset{¯}{x}}_{j}-{\overset{¯}{x}}_{j^{\prime }}\right)\left(\Delta_{i,j}-\Delta_{i^{\prime },j^{\prime }}\right)\right)\\ & =\frac{2}{{\left(2n\right)}^{2}d\left(d-1\right)}\sum_{j=1}^{d-1}\sum_{j^{\prime }=j+1}^{d}\sum_{i=1}^{2n}\sum_{i^{\prime }=1}^{2n}\left({\left({\overset{¯}{x}}_{j}-{\overset{¯}{x}}_{j^{\prime }}\right)}^{2}+{\left(\Delta_{i,j}-\Delta_{i^{\prime },j^{\prime }}\right)}^{2}\right)\\ & =\frac{2}{d(d-1)}\sum_{j=1}^{d-1}\sum_{j^{\prime }=j+1}^{d}{\left({\overset{¯}{x}}_{j}-{\overset{¯}{x}}_{j^{\prime }}\right)}^{2}+\frac{2}{{\left(2n\right)}^{2}d\left(d-1\right)}\sum_{j=1}^{d-1}\sum_{j^{\prime }=j+1}^{d}\sum_{i=1}^{2n}\sum_{i^{\prime }=1}^{2n}\left(\Delta_{i,j}^{2}+\Delta_{i^{\prime },j^{\prime }}^{2}-2\Delta_{i,j}\Delta_{i^{\prime },j^{\prime }}\right)\\ & =\frac{2}{d(d-1)}\sum_{j=1}^{d-1}\sum_{j^{\prime }=j+1}^{d}{\left({\overset{¯}{x}}_{j}-{\overset{¯}{x}}_{j^{\prime }}\right)}^{2}+\frac{2}{{\left(2n\right)}^{2}d\left(d-1\right)}\sum_{j=1}^{d-1}\sum_{j^{\prime }=j+1}^{d}\sum_{i=1}^{2n}\sum_{i^{\prime }=1}^{2n}\left(\Delta_{i,j}^{2}+\Delta_{i^{\prime },j^{\prime }}^{2}\right)\\ & =\frac{2}{d(d-1)}\sum_{j=1}^{d-1}\sum_{j^{\prime }=j+1}^{d}{\left({\overset{¯}{x}}_{j}-{\overset{¯}{x}}_{j^{\prime }}\right)}^{2}+\frac{2}{2nd(d-1)}\left(\sum_{j=1}^{d-1}\sum_{j^{\prime }=j+1}^{d}\sum_{i=1}^{2n}\Delta_{i,j}^{2}+\sum_{j=1}^{d-1}\sum_{j^{\prime }=j+1}^{d}\sum_{i^{\prime }=1}^{2n}\Delta_{i^{\prime },j^{\prime }}^{2}\right)\\ & =\frac{2}{d(d-1)}\sum_{j=1}^{d-1}\sum_{j^{\prime }=j+1}^{d}{\left({\overset{¯}{x}}_{j}-{\overset{¯}{x}}_{j^{\prime }}\right)}^{2}+\frac{2}{2nd}\sum_{j=1}^{d}\sum_{i=1}^{2n}\Delta_{i,j}^{2}\\ & =2{\hat{V}}_{H,B}+2{\tilde{V}}_{H,W} \end{array}$$

and at Hardy–Weinberg equilibrium,

(8) $$S_{H,B}=2{\hat{V}}_{A,B}+4{\tilde{V}}_{A,W}$$

where ${\hat{V}}_{H,B}$ is the unbiased estimator of the variance in breeding values among the haplotypes of individuals from different groups, and ${\tilde{V}}_{H,W}$ and ${\tilde{V}}_{A,W}$ are biased estimators (i.e., division by 2*nd* or *nd* instead of 2*nd*−*d* or *nd*−*d*), respectively, of the within‐group variances $V_{H,W}$ and $V_{A,W}$ (for a similar derivation see Goldstein, Linares, Cavalli‐Sforza, & Feldman, 1995). Combining Equations (5) and (7) gives

(9) $$E\left\{{\hat{V}}_{A,W}\right\}=\tau_{W}\sigma_{m}^{2}.$$

Combining Equations (6) and (8) and assuming *n* is large gives

(10) $$\frac{1}{2}E\left\{{\hat{V}}_{A,B}\right\}+E\left\{{\hat{V}}_{A,W}\right\}\approx \tau_{B}\sigma_{m}^{2}.$$

Additionally, because the mean coalescence time of pairs of alleles from the collection of groups sampled, τ, can be defined as a weighted sum of the within‐group and between‐group coalescence times for the sample (Slatkin, 1995),

(11) $$\tau =\frac{2n(d-1)}{2nd-1}\tau_{B}+\frac{2n-1}{2nd-1}\tau_{W},$$

if, as above, *n* is large

(12) $$\frac{d-1}{2d}E\left\{{\hat{V}}_{A,B}\right\}+E\left\{{\hat{V}}_{A,W}\right\}\approx \tau \sigma_{m}^{2}.$$

We can now express ${\hat{Q}}_{\mathrm{ST}}^{\mathrm{PBS}}$ and ${\hat{Q}}_{\mathrm{ST}}^{\mathrm{RB}}$ in terms of coalescence times. For ${\hat{Q}}_{\mathrm{ST}}^{\mathrm{PBS}}$, combining Equation (1) with Equations (9) and (10) gives

$$\begin{array}{cc} & \frac{E\{{\hat{V}}_{A,B}\}}{E\left\{{\hat{V}}_{A,B}\right\}+2E\left\{{\hat{V}}_{A,W}\right\}}\\ & =\frac{\frac{1}{2}E\left\{{\hat{V}}_{A,B}\right\}+E\left\{{\hat{V}}_{A,W}\right\}-E\left\{{\hat{V}}_{A,W}\right\}}{\frac{1}{2}E\left\{{\hat{V}}_{A,B}\right\}+E\left\{{\hat{V}}_{A,W}\right\}}\\ & =\frac{\tau_{B}-\tau_{W}}{\tau_{B}}. \end{array}$$

For ${\hat{Q}}_{\mathrm{ST}}^{\mathrm{RB}}$, combining Equation (3) with Equations (9) and (12) gives

$$\begin{array}{cc} & \frac{E\{{\tilde{V}}_{A,B}\}}{E\left\{{\tilde{V}}_{A,B}\right\}+2E\left\{{\hat{V}}_{A,W}\right\}}\\ & =\frac{\frac{d-1}{d}E\left\{{\hat{V}}_{A,B}\right\}}{\frac{d-1}{d}E\left\{{\hat{V}}_{A,B}\right\}+2E\left\{{\hat{V}}_{A,W}\right\}}\\ & =\frac{\frac{d-1}{2d}E\left\{{\hat{V}}_{A,B}\right\}+E\left\{{\hat{V}}_{A,W}\right\}-E\left\{{\hat{V}}_{A,W}\right\}}{\frac{d-1}{2d}E\left\{{\hat{V}}_{A,B}\right\}+E\left\{{\hat{V}}_{A,W}\right\}}\\ & =\frac{\tau -\tau_{W}}{\tau }. \end{array}$$

Therefore, the two $Q_{\mathrm{ST}}$ estimators have somewhat different interpretations in terms of coalescence times. In summary, ${\hat{Q}}_{\mathrm{ST}}^{\mathrm{PBS}}$ implies

$$Q_{\mathrm{ST}}=\frac{\tau_{B}-\tau_{W}}{\tau_{B}}\;\text{and}$$

${\hat{Q}}_{\mathrm{ST}}^{\mathrm{RB}}$ implies

$$Q_{\mathrm{ST}}=\frac{\tau -\tau_{W}}{\tau }.$$

## 4 Relationships Between $Q_{\mathrm{ST}}$ and $F_{\mathrm{ST}}$ Estimators

As discussed by Slatkin (1993, 1995), a similar situation occurs for $F_{\mathrm{ST}}$ in that the different estimators have somewhat different interpretations in terms of coalescence times. For example, Weir and Cockerham (1984)'s $\hat{\theta }$ and Lynch and Crease (1990)'s $N_{\mathrm{ST}}$ imply

$$F_{\mathrm{ST}}=\frac{\tau_{B}-\tau_{W}}{\tau_{B}},$$

and Nei (1973)'s $G_{\mathrm{ST}}$ and Slatkin (1995)'s $R_{\mathrm{ST}}$ imply

$$F_{\mathrm{ST}}=\frac{\tau -\tau_{W}}{\tau }.$$

Therefore, $\hat{\theta }$ and $N_{\mathrm{ST}}$ correspond to ${\hat{Q}}_{\mathrm{ST}}^{\mathrm{PBS}}$, and $G_{\mathrm{ST}}$ and $R_{\mathrm{ST}}$ correspond to ${\hat{Q}}_{\mathrm{ST}}^{\mathrm{RB}}$.

## 5 Relating the Results to PreviousWork

Whitlock (2008) noted that, if the character and DNA marker are evolving neutrally, ${\hat{Q}}_{\mathrm{ST}}^{\mathrm{PBS}}$ and $\hat{\theta }$ are expected to match, which is consistent with the results presented here, and he used simulations to demonstrate this correspondence in a 10 deme island model. However, according to Whitlock (1999), ${\hat{Q}}_{\mathrm{ST}}^{\mathrm{PBS}}$ implies $Q_{\mathrm{ST}}=\left(\tau -\tau_{W}\right)/\tau$, which is only accurate when *d* is large (many groups have been sampled). As shown here, the more general result (i.e., applicable to an arbitrary number of sampled groups) is that ${\hat{Q}}_{\mathrm{ST}}^{\mathrm{PBS}}$ implies $Q_{\mathrm{ST}}=\left(\tau_{B}-\tau_{W}\right)/\tau_{B}$.Whitlock (1999)'s result holds when *d* is large, because the limit of τ as the number of sampled groups goes to infinity is $\tau_{B}$ (see Equation (11)).

It is also instructive to relate the coalescent‐based results presented here and in Whitlock (1999) to classic results of evolutionary quantitative genetics. Imagine a simple split (i.e., without gene flow) *t* generations in the past of an ancestral group into two descendant groups, both of which have the same effective population size as the ancestral group. Under this scenario, if *n* is large, $\tau \approx \tau_{W}+t/2$ (Slatkin, 1995), and according to formulas in Whitlock (1999), ${\hat{V}}_{A,B}$ is expected to be $2\sigma_{m}^{2}\left(\tau -\tau_{W}\right)$. Combining these results for τ and ${\hat{V}}_{A,B}$ leads to the expectation that ${\hat{V}}_{A,B}$ will be $\sigma_{m}^{2}t$ for a simple split into two groups, which differs by a factor of two from classic results (Lande, 1979; Lynch & Hill, 1986; Turelli, Gillespie, & Lande, 1988). This contradiction can be resolved by recognizing that the more general result, from Equations (10) and (12), is

$$\begin{array}{cc} E\{{\hat{V}}_{A,B}\} & =2\sigma_{m}^{2}\left(\tau_{B}-\tau_{W}\right)\\ & =\frac{d}{d-1}2\sigma_{m}^{2}\left(\tau -\tau_{W}\right), \end{array}$$

which leads to the expectation, consistent with classic results, that ${\hat{V}}_{A,B}$ will be $2\sigma_{m}^{2}t$ for a simple split into two, or more, groups.

## 6 Conclusions

A variety of estimators have been developed for $Q_{\mathrm{ST}}$ and $F_{\mathrm{ST}}$, and they are not equivalent, having somewhat different interpretations in terms of coalescence times for neutrally evolving characters. Particular estimators are not inherently “better" than the others, but when the number of groups that have been sampled is small, it is important to match estimators appropriately when comparing $Q_{\mathrm{ST}}$ to $F_{\mathrm{ST}}$ (see also Whitlock, 2008). When observations come from a large number of groups, all of the estimators converge, so proper matching is less critical.

## Conflict of Interest

None declared.
